# Comparative Pharmacokinetic Study of Three Major Bioactive Components in the Normal and Different Courses of Liver-Stagnation and Spleen-Deficiency Syndrome Depressive Rats after Intragastric Administration of Zhi-Zi-Hou-Po Decoction

**DOI:** 10.1155/2022/8657616

**Published:** 2022-03-24

**Authors:** Kaiwen Luo, Mengdie Wang, Xiangxiang Pan, Yadong Xing

**Affiliations:** School of Pharmacy, Bengbu Medical University, Bengbu 233030, China

## Abstract

To reveal the pharmacokinetic process of narirutin, naringin, and honokiol in normal and different courses of liver-stagnation and spleen-deficiency syndrome depressive rats after intragastric administration of Zhi-Zi-Hou-Po decoction (ZZHPD), a rapid ultra-high performance liquid chromatography coupled to tandem mass spectrometry (UHPLC-MS/MS) method was proposed in this study. Chronic unpredictable mild stress (CUMS) rat was employed as the depression model. Acetonitrile solution containing 0.1% acetic acid and methyl alcohol-water (50 : 50, *v*/*v*) was chosen as the protein precipitant and redissolve solution severally; a Shim-pack GISS C_18_ column coupled with 0.1% aqueous acetic acid-acetonitrile in gradient elution was employed to separate the mixed constituents in plasma. The WinNonLin software (version 6.1) was chosen as the analytical tool for the pharmacokinetic parameters. The results indicated that compared with rats in the control group, the sucrose preference, scores in the open-field test, and the concentration of 5-HT in plasma of rats in CUMS and CUMS + ZZHPD treatment groups were lower, while the immobility time in the forced swimming test of rats in these two groups was longer, which implied that the depression model was successful. These behavioral and biochemical indexes of rats above in the CUMS + ZZHPD treatment group were improved after oral administration of ZZHPD, which indicated that the antidepressant effect of ZZHPD was definite. The UHPLC-MS/MS method was stabilized, sensitive, and exclusive, and the extraction recovery and matrix effect of three analytes were all above 89%. The *T*_max_, AUC, and C_max_ of three ingredients in CUMS-induced depression rats were significantly larger than control rats, while these pharmacokinetic parameters in CUMS + ZZHPD treatment rats were decreased significantly compared with CUMS-induced depression rats, which may relate with the changes in physiological function of the gastrointestinal tract and liver in CUMS-induced liver-stagnation and spleen-deficiency syndrome depressive rats. This study provided important information for the clinical rational use of ZZHPD in antidepressant treatment.

## 1. Introduction

Nowadays, the prevalence of depression is increasing year by year due to the life stress increasing of people. The World Health Organization (WHO) claims that depression will rise to the top of the world's disease burden by 2030 [1]. Currently, the pharmacological mechanism of antidepressant chemical drugs commonly used in clinical involves the reuptake inhibition or receptor block of neurotransmitters, such as 5-hydroxytryptamine (5-HT) and norepinephrine (NE), resulting in fight or relief of depressive symptoms, which may generate different degrees of toxic side effects on patients after medication [2]. Therefore, it is an urgent job to seek new alternative drugs which are more effective and have lower side effects.

Traditional Chinese medicine formula (TCMF), which could exert synergistic therapeutic efficacies on multiple targets with multiple constituents, plays an important role in the clinical to prevent and treat depression in China and some Asian countries. Zhi-Zi-Hou-Po decoction (ZZHPD) was originally recorded in Shang-Han-Lun (Treatise on Febrile Caused by Cold) in the Han Dynasty of ancient China. It is one of the classical formulas in treating depression in China, which contains three medicinal herbs: *Magnolia officinalis* Rehd. Et Wils. (which is called “Hou-po” in China), *Gardenia jasminoides* Ellis (which is called “Zhi-zi” in China), and *Citrus aurantium* L. (which is called “Zhi-shi” in China). For now, there are many researches that focus on the chemical profiling [3], metabolic profile [4, 5], and pharmacological mechanisms [6, 7], and comparative pharmacokinetic study [8] and potential toxicity mechanisms [9, 10] of ZZHPD have been done. The results of these studies indicated that the possible antidepressant mechanism of ZZHPD was to enhance the monoaminergic system and promote hippocampal neurogenesis, lignans, and flavonoids the possible effective components, while the potential toxicity is related to the change in the dosage or component content of ZZHPD.

Pharmacokinetic studies on TCMF are important for clinical efficacy evaluation, medication guidance, and the further development of TCMF [11]. Compared with the normal one, the pharmacokinetic parameters of TCMF in disease life entity would be changed due to the changes in intestinal flora, the humoral microenvironment, and enzyme activity [8]. Then these vital signs would be called-back after treatment [7]. Meanwhile, the pharmacokinetic profiles of bioactive ingredients of TCMF may change. Geniposide is one of the characteristic iridoid in Zhi-zi, naringin, narirutin, hesperidin, and neohesperidin are the characteristic flavonoids in Zhi-shi, and magnolol and honokiol are the main bioactive lignans in Hou-po. These chemical components were claimed to possess multiple activities. Geniposide [12] and hesperidin [13] were claimed to exert therapeutic efficacy on a depressive mice model. Neohesperidin could possess a neuroprotective effect on primary hippocampal cells [14]. Naringin could relieve depressive symptoms [15] and protect neuro [16]. Narirutin could produce an antidepressant-like effect [17] and attenuate alcoholic liver disease [18]. Honokiol could promote nonrapid eye movement sleep [19], possess antidepressant-like effect, apoptotic effect [20], and antihepatic fibrosis effect [21]. Magnolol could alleviate depressive-like behaviors in depressive mice [22]. The comparative pharmacokinetics of geniposide, hesperidin, neohesperidin, and magnolol between normal and depression rat have been reported [8], so further studying the pharmacokinetics of narirutin, naringin, and honokiol in normal and different courses of depression rats is so important to the research of the pharmacodynamic material basis and antidepressant mechanism of ZZHPD. The syndrome of liver-stagnation and spleen-deficiency patients, which is described in traditional Chinese medicine theory, is in accord with the symptoms of depression in clinical [23, 24]. In addition, the chronic unpredictable mild stress (CUMS) model, which is one of the classic models of depression, has been proven very similar to the liver-stagnation and spleen-deficiency syndrome models in the aspect of body weight change, mental state, and gastrointestinal symptoms [24]. Hence, the CUMS depression model was chosen to investigate the antidepressant effect and the pharmacokinetic parameters of ZZHPD in this study.

In this study, an UHPLC-MS/MS method, which was selective, accurate, and efficient, was developed to detect naringin, narirutin, and honokiol synchronously. The pharmacokinetic profiles of these three ingredients in normal, depressed, and cured depression rats after intragastric administration of ZZHPD were compared. It was credible that the results of this study could provide helpful information for the quality control and modern clinical application of ZZHPD.

## 2. Experimental

### 2.1. Chemicals and Reagents

**Figure 1:**
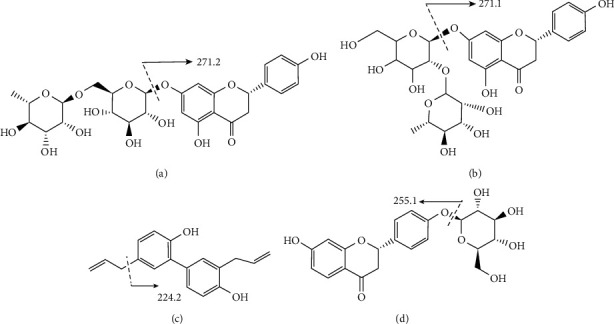
The chemical structures and fragmentations of narirutin (a), naringin (b), honokiol (c), and liquiritin (IS) (d).

Paroxetine hydrochloride (PX) was purchased from Sigma-Aldrich (St. Louis, MO, USA). Naringin (lot no. MUST-20041803), narirutin (lot no. MUST-20111105), honokiol (lot no. MUST-20032205), and liquiritin (lot no. MUST-20052110) were purchased from Must Biological Technology Co. Ltd. (Chengdu, China). The chemical structures and fragmentations of these four chemical components are shown in Figure 1. The purity of these reference substances above was all above 98%.

*Gardenia jasminoides* Ellis (180821, Jiangxi, China), *Citrus aurantium* L. (180921, Jiangxi, China), and *Magnolia officinalis* Rehd. et Wils. (180913, Sichuan, China) were purchased from Bozhou Yonggang Decoction Piece Factory Co. Ltd. (Bozhou, China). Formic acid and acetic acid were purchased from Anpu Experimental Technology Co., Ltd. (Shanghai, China). Methanol and acetonitrile were purchased from Merck Reagent Co., Ltd (Darmstadt, Germany). Other reagents were purchased from McLean Biochemical Technology Co., Ltd (Shanghai, China).

### 2.2. Animals

Sprague Dawley rats (male) were obtained from the Qinglong Mountain Animal Breeding Farm (Nanjing, China) and raised in the Experimental Animal Center of Bengbu Medical University (Bengbu, China). The rats' age was 3-4 weeks and they weigh 100 ± 20 g. The experimental protocol and animal studies were authorized by the Animal Care and Use Committee of Bengbu Medical University (approval no. A-2020-034) and conformed to the Guidelines for the Use of Laboratory Animals (National Research Council).

### 2.3. Animal Dosing and Sample Collection

During the first week (adaptive phase), rats were freed to eat food and drink purified water, and the feeding condition was set as follows: humidity 40–70%, breeding room temperature 20–25°C, and light/dark cycle 12 h. The rats in all groups were jejunitas for 12 h before the experiment. The process of animal experimentation can be seen in Figure S1. On day 1 of week 2, rats were randomly divided into four groups and eight in each, on the basis of the results of a sucrose preference test (SPT). From day 2 of week 2, the stressors of CUMS were employed to exert stress on rats in CUMS, CUMS + ZZHPD, and CUMS + paroxetine hydrochloride (PX) groups for 8 weeks, and each rat was housed in each solitary cage. The forms of stressors were referred to our previous study [25], including water deprivation (24 hr), noise stress (1 hr), physical restraint (2 hr), forced swimming in ice-water (10 min), reversed light/dark cycle (24 hr), food deprivation (24 hr), clipping tail (5 min), cage tilting at 45°(24 hr), horizontal oscillation (20 min), and soiled bedding (300 mL water in 150 g sawdust bedding 20 hr). Rats in modeling groups including CUMS, CUMS + PX, and CUMS + ZZHPD were subjected to one or two styles of stressors randomly in a single day until day 7 of week 8. There was no treatment brought to bear on rats in the control group in the same time. As per the therapeutic schedule, rats in groups of CUMS + PX and CUMS + ZZHPD were intragastrically administered by ZZHPD (8.5 g/kg/d) and PX (2.1 mg/kg/d), respectively, from day 1 of week 5 to day 7 of week 8. The dosage was converted from the human dosage (text S1). Rats in groups of control and CUMS were given the equal volume of distilled water during this experiment period. To evaluate the depressive state of rats, their multifarious behavioral characteristics and related plasma biochemical index were tested in weeks 5 and 9.

For the pharmacokinetic study, rats in control, the rats in groups of CUMS + ZZHPD and CUMS were intragastrically administered by ZZHPD on a dose of 8.5 g/kg/d at day 1 of week 10. Then blood was collected by a heparinized tube via the caudal vein of each rat at 0, 0.083, 0.167, 0.333, 0.5, 0.667, 1, 2, 3, 4, 6, 8, 12, and 24 h after administration. To avoid the influence of long-term ZZHPD treatment on the plasma drug concentration of rats in the CUMS + ZZHPD group, there was a withdrawal period for one week between week 8 and week 10. To acquire the plasma samples, the blood was oscillated and centrifuged, and then the liquid supernatant was collected and stored at −20°C until analysis.

### 2.4. LC-MS Conditions

For biologic sample analysis, an UHPLC-MS/MS system consisting of Nexera UHPLC LC-30A (Shimadzu Corporation, Kyoto, Japan) and AB Sciex Qtrap 5500 (Applied Biosystems, Foster City, CA, USA) was used. For LC-30A, it was equipped with DGU-20A_5_ vacuum degasser, 30AD binary pump, CTO-30A column oven, and SIL-30AC autosampler. For Qtrap 5500, it was equipped with an electrospray ionization (ESI) source. The mobile phase, which was formed from phase A (acetic acid: water = 0.1 : 100, *v*/*v*) and phase B (acetonitrile), was set as a linear elution gradient: 0 min, 15% B; 7 min, 15% B; 8 min, 70% B; 10 min, 85% B; 11 min, 85% B; 11.5 min, 15% B; 12 min, 15% B. These analytes were separated on a Shim-pack GISS C_18_ column (Shimadzu Corporation, Kyoto, Japan) at 35°C. The specifications of the column were 50 mm × 2.1 mm, 1.9 *μ*m. The injection volume was set as 10 *μ*L. QqQ/MS controlled by Analyst™ version 1.5.2 (Applied Biosystems) was utilized for data acquisition.

For optimizing the MS parameters, the standard solutions of all analytes, including three ingredients and IS, were injected directly via a syringe pump. The common parameters of analytes were set as follows: the mass detector was operated in negative ionization mode, the source temperature was 550°C, the ion spray voltage was −4500 V, the curtain gas was 25 psi, and the nebulizing gas (N_2_) was 55 psi, as well as the auxiliary gas. The other quantitative parameters are listed in Table 1, including precursor ion/product ion, declustering potential (DP), collision energy (CE), entrance potential (EP), and cell exit potential (CXP).

### 2.5. Behavioral Tests and Biochemical Analysis

For the assessment of the depression model and the antidepressant efficacy of positive medicine and ZZHPD, behavioral tests and biochemical analysis were employed. Referring to our previous study [25], the SPT, forced swimming test (FST), open-field test (OFT), and the concentration detection of 5-hydroxytryptamine (5-HT) in plasma were as follows: In simple terms, the ratio of 1% sucrose solution consumption and total liquid consumption (1% sucrose solution and pure water) of each rat in 1 h was regarded as the sucrose preference degree in SPT, the activity level of each rat in a black box without a top (80 × 80 × 40 cm) in 3 min was counted as the OFT score, and the immobility time of rats in a bucket filled with water in 4 min was recorded as the forced swimming test score. The enzyme-linked immunosorbent assay (ELISA) kits (mlbio, Shanghai, China) were used to detect the concentration of 5-HT in plasma.

### 2.6. ZZHPD Sample Preparation

The preparation process of the ZZHPD sample was referred to our previous study [4]. In simple terms, the powder of ZZ (9 g), HP (62.4 g), and ZS (10 g) were boiled and filtered 3 times. The volumes of water were 1 : 10, 1 : 8, and 1 : 5 for each time. Before the first boiling, the mixed powder was soaked in water for 30 min. The decoction filtrate of three times was mixed and enriched to 1.628 g/mL (crude drug concentration). For UHPLC-MS/MS detection, 1.5 mL extract and 1.5 mL of water were mixed, and then absolute ethyl alcohol was added until the total volume was up to 10 mL. After oscillation and centrifugation, the supernate was collected and diluted to an appropriate concentration.

### 2.7. Preparation of Plasma Samples

After being thawed at room temperature, an aliquot of 100 *μ*L plasma was precipitated with 200 *μ*L acetonitrile which contained IS (concentration of 2.5 ng/mL) and 0.1% acetic acid (*v*/*v*). After vortex-blending for 3 min and centrifugation at 11000 rpm for 5 min, the supernate was collected and transferred to a new Eppendorf tube (specification 1.5 mL) and freeze-dried by a freezing centrifugal dryer (model no. LNG-96, Hunan Hukang Centrifuge Co. Ltd, Changsha, China). For UHPLC-MS/MS analysis, a volume of 100 *μ*L methyl alcohol-water (50 : 50, *v*/*v*) was added to the dried residue. After vortex-mixing and centrifugation at 12000 rpm for 10 min, the supernatant liquid was transferred to an injection vial with a lining tube. The injection volume for each time was 10 *μ*L.

### 2.8. Standard and Quality Control Solution

To prepare the stock solutions, the reference substances of narirutin, naringin, honokiol, and liquiritin were weighed critically and then dissolved to a constant volume of 10 mL by methyl alcohol. The concentrations of analytes and IS stock solutions were as follows: narirutin 552 *μ*g/mL, naringin 632 *μ*g/mL, honokiol 132 *μ*g/mL, and IS 413 *μ*g/mL. For acquiring mixed standard solutions, the first three of these stock solutions above were taken 60, 200, and 250 *μ*L severally and then mixed and diluted with methanol, finally a series of standard working solutions at concentration of 1.0, 1.7, 3.3, 6.6, 16.6, 33.1, 66.2, 165.6, and 331.2 ng/mL for narirutin, 3.8, 6.3, 12.6, 25.3, 63.2, 126.4, 252.8, 632.0, and 1264.0 ng/mL for naringin, and 1.0, 1.7, 3.3, 6.6, 16.5, 33.0, 66.0, 165.0, 330.0 ng/mL for honokiol, which were used for the calibration curve. The concentrations of these analytes in low, middle, and high quality control (QC) solutions were as follows: narirutin 2.5, 20, and 265 ng/mL; naringin 9.5, 76, and 1011.2 ng/mL; honokiol 2.5, 20, and 264 ng/mL. All the solutions above were stored at 4°C before use.

### 2.9. Method Validation

Referring to USA-FDA Bioanalytical Method Validation Guidance [26], the specificity, recovery, stability, precision, accuracy, linearity, and matrix effect of the UHPLC–MS/MS method proposed in this study were fully validated.

For specificity evaluation, plasma samples acquired after intragastric administration of ZZHPD, blank plasma, and blank plasma mixed with the three analytes and IS were analyzed by LC-MS to judge endogenous interference.

For linearity evaluation, an aliquot of 200 *μ*L plasma, 100 *μ*L standard working solution, and 100 *μ*L IS standard solution (1.2 ng/mL) were mixed, and then the mixture was pretreated according to the method mentioned in “sample preparation.” The calibration curve was plotted by weighed least-squares linear regression using 1/*x*^2^ as the weighting factor. The theoretical concentrations of analytes were defined as the *x*-axis, and the peak area ratios of each analyte to IS were defined as the *y-*axis severally. The lower limit of quantification (LLOQ) of each analyte was chosen as the lowest concentration on each calibration curve. The acceptable accuracy and precision, which were evaluated by relative error (RE, within ±20%) and relative standard deviation (RSD, <20%), respectively, were based on the analysis of one sample for six replicates.

Precision and accuracy were verified by analysis of six replicated blank plasma spiked with three levels of QC samples at low, middle, and high concentrations on the same day and on three successive days. The acceptable range for RSD of intraday and interday precision is <15%, while the acceptable range for RE of accuracy is −15%∼15%.

To evaluate the stability of bio-samples in different storage conditions, three levels of QC solutions in plasma were analyzed at room temperature for four hours, at 4°C (placed in automatic sampler) for twelve hours, after freeze-thaw for three times, and at −20°C for 14 days. The analytes could not be identified as stable until the accuracy bias was within ±15%.

For extraction recovery evaluation, the peak area ratio of analytes in six repetitive extracted samples and postextracted samples spiked with QC solutions was employed. To appraise the matrix effect, the peak area ratio of analytes in six repetitive postextracted samples spiked with QC solutions and pure standard solutions at the same concentration was adopted. The reasonable range should be 85%∼115%.

### 2.10. Pharmacokinetic Study and Data Analysis

As described in part of “animal experiments,” the plasma samples in the control, CUMS, and CUMS + ZZHPD treatment groups (*n* = 6) were acquired for pharmacokinetic study. By concentration determination and calculation of three active ingredients in ZZHPD, the dosages of narirutin, naringin, and honokiol for rats were 6.525, 97.092, and 19.58 mg/kg, respectively. For LC-MS/MS analysis, these plasma samples were disposed of by the pretreatment method above, and the WinNonLin software (version 6.1) was used for the calculation of the pharmacokinetic parameters by noncompartmental analysis. Meanwhile, to compare the pharmacokinetic parameters, including *C*_max_, *T*_max_, t_*1*/*2*_, AUC_0-*t*_, and AUC_0-∞_, of CUMS-induced liver-stagnation and spleen-deficiency syndrome depressive rats in different courses, SPSS 19.0 software (SPSS Inc., Chicago, IL, USA) was employed. The data were presented as the mean ± SD, and the *p* value less than 0.05 was regarded as statistically significant.

## 3. Results and Discussion

### 3.1. Evaluation of Depression Model and ZZHPD Efficacy

**Figure 2:**
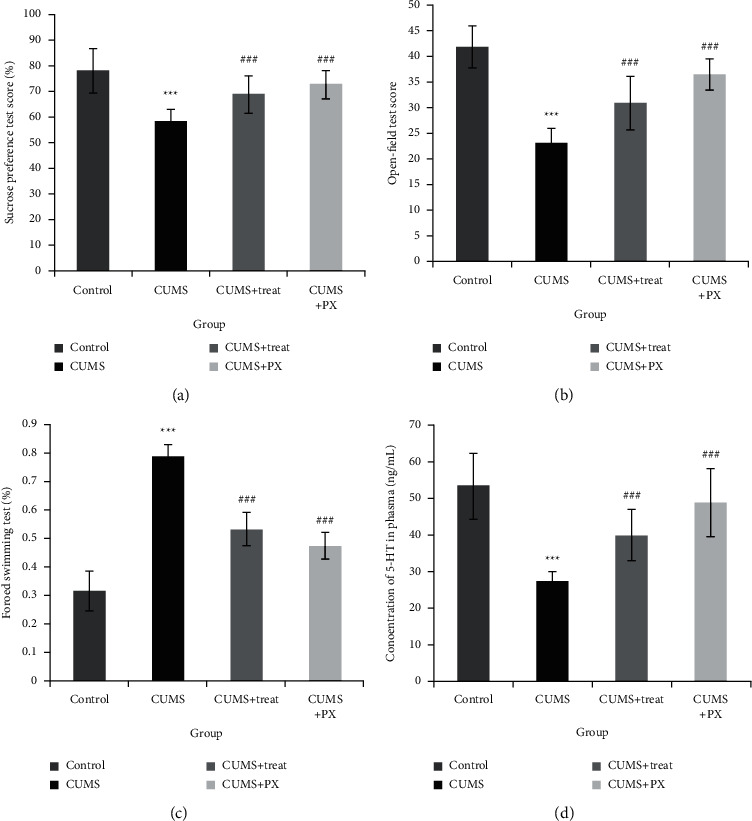
The results of rat behavioral tests and biochemical analysis in week 9 (*n* = 6). The sucrose preference test (SPT) score (a), open-field test (OFT) score (b), and concentration of 5-HT in plasma (d) were lower, and the forced swimming test (FST) score (c) was higher in the CUMS group compared with the control group. While the SPT score (a), OFT score (b), and concentration of 5-HT in plasma (d) were higher, FST score was lower in CUMS + treat (ZZHPD treatment) group and CUMS + PX group compared with CUMS group. ^*∗∗∗*^*p* < 0.001 different from control group; ^##^*p* < 0.01 ^###^*p* < 0.001 different from CUMS group (measured by one-way ANOVA).

As for the evaluation indexes of the depression model and antidepressant efficacy of ZZHPD, the results of SPT, OFT, FST, and the concentration of 5-HT in plasma in week 9 are listed in Figure 2. It could be seen that compared with rats in the control group, the sucrose preference was lower (Figure 2(a)), scores in OFT were lower (Figure 2(b)), immobility time was longer (Figure 2(c)), and the concentration of 5-HT in plasma was lower (Figure 2(d)) in the CUMS group, which implied that the depression model was successful. After intragastric administration of ZZHPD for four weeks, rats in the CUMS + ZZHPD treatment group showed obvious upregulation on sucrose preference in SPT (Figure 2(a)), score in OFT (Figure 2(b)), and the concentration of 5-HT in plasma (Figure 2(d)) and downregulation on immobility time in FST (Figure 2(c)) compared with rats in the CUMS group in week 9, by which the antidepressant effect of ZZHPD was proved. All the results were measured by one-way ANOVA (SPSS 19.0 software, Chicago, IL, USA).

### 3.2. Optimization of Plasma Samples Preparation

To investigate the preprocessing method of plasma samples, liquid-liquid extraction (LLE), solid phase extraction (SPE) and protein precipitation were employed and compared. For each method, the condition parameters were optimized, including the type and volume ratio of extracting solvent (ethyl acetate, chloroform) in LLE, the pH of eluent (methanol) and cubage of the C_18_ column in SPE, and the type and volume ratio of precipitate reagent (methanol and acetonitrile), as well as the type and concentration of modifying agents (formic acid and acetic acid) in the precipitate reagent in protein precipitation. In consideration of the extraction recovery, stability, and matrix effect synthetically, protein precipitation was chosen as the preprocessing method. The precipitate reagent was acetonitrile containing 0.1% acetic acid (*v*/*v*), the volume ratio of plasma to acetonitrile was 1 : 2 (*v*/*v*).

### 3.3. Optimization of UHPLC–MS/MS Conditions

The mobile phase conditions could influence the peak symmetry, analytical time, and ionization of the analytes. In this study, the type, modifying agent, and the gradient of mobile phase were optimized. Acetonitrile showed better separation efficiency than methyl alcohol. For peak symmetry of analytes, acetic acid was the better modifying agent than formic acid, and the best concentration was 0.1% (*v*/*v*). In view of resolution and retention time, the time program of the mobile phase was set as gradient elution. To eliminate the interference of endogenous components, potential metabolites, and the isomeride (narirutin and naringin), gradient elution program was optimized as follows: phase A (acetic acid: water = 0.1 : 100, *v*/*v*) and phase B (acetonitrile), 0 min, 15% B; 7 min, 15% B; 8 min, 70% B; 10 min, 85% B; 11 min, 85% B; 11.5 min, 15% B; 12 min, 15% B.

**Figure 3:**
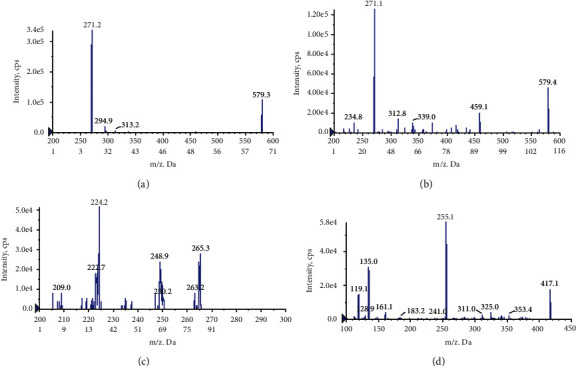
Full scan product ion spectra of narirutin (a), naringin (b), honokiol (c), and IS (d) in negative ionization mode.

The sensitivity of the analysis method was closely connected with the MS conditions. These analytes displayed higher sensitivity in the negative ion mode. For narirutin, naringin, honokiol, and IS, [M-H]^–^ was the most abundant quasimolecular ions, while the product ions were *m*/*z* 271.1, 271.2, 224.2, and 255.1 severally. For the multiple reaction monitoring (MRM) transitions of narirutin, naringin, honokiol, and IS, the optimized scope of collision energy values was set from 10 to 60 eV, resulting the best values for narirutin, naringin, honokiol, and IS were 33.4, 43.7, 30.5, and 25.8, respectively, under which conditions the ion intensity of major fragment ions was high enough and the interference of other fragmentations was low. The DP, EP, and CXP of these analytes were also optimized, which is listed in Table 1, and the full scan product ion spectra of narirutin (a), naringin (b), honokiol (c), and IS (d) in negative ionization mode are shown in Figure 3.

### 3.4. Method Validation

#### 3.4.1. Specificity

**Figure 4:**
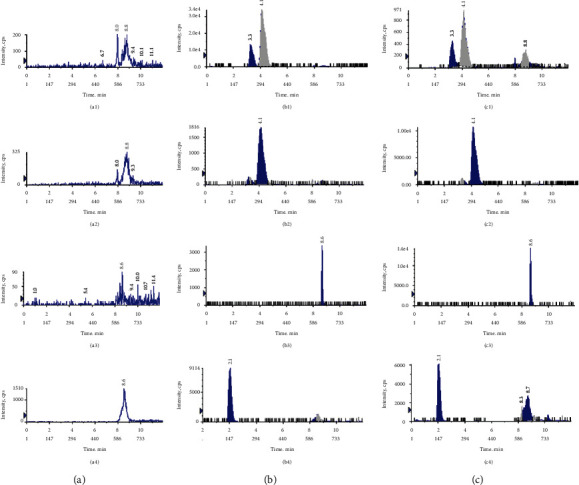
Representative chromatograms of narirutin (1), naringin (2), honokiol (3), and IS (4) in negative ionization mode in blank plasma (a), blank plasma sample spiked with the analytes (b), and plasma sample from a normal rat 1 h after oral administration of ZZHPD (c).

To assess the specificity, endogenous interferences near the retention times of these analytes were selected as the indicators. The representative chromatograms of narirutin, naringin, honokiol, and IS in negative ionization mode in the blank plasma sample, blank plasma sample spiked with these analytes, and plasma sample from a normal rat 1 h after intragastric administration of ZZHPD are exhibited in Figure 4. It could be seen that there were no obvious interfering peaks near the peaks of these analytes in the chromatograms of blank plasma samples, spiked samples, and plasma samples acquired after oral administration.

#### 3.4.2. LLOQ and Linearity

The LLOQ of the UHPLC-MS/MS method for narirutin, naringin, and honokiol was 1, 3.8, and 1 ng/mL, respectively. Furthermore, the linear ranges for the three analytes were as follows: 1.0–331.2 for narirutin, 3.8–1264.0 for naringin, and 1.0–330.0 for honokiol, the linear regression equations and sample correlation coefficients (*r*) for ingredients above were list in Table 2, which met requirements of pharmacokinetic study. The standard curves of narirutin (a), naringin (b), and honokiol (c) (*r* > 0.99) are shown in Figure S2.

#### 3.4.3. Precision and Accuracy

The intraday and interday precision of the three analytes were as follows: RSD was below 2.11% and RE was between −4.16% and 6.16 for narirutin, RSD was below 2.01% and RE was between −5.21 and 7.91% for naringin, and RSD was below 2.39% and RE was between −7.28 and 8.14% for honokiol, which indicated that this method possessed good precision and accuracy. All the results of precision and accuracy in this study were exhibited in Table S1.

#### 3.4.4. Matrix Effect and Extraction Recovery

For narirutin, naringin, and honokiol at three concentration levels, the recovery ranges were 92.25 ± 4.21–98.23 ± 5.36, 89.62 ± 2.76–99.69 ± 5.56, and 91.92 ± 1.03–104.71 ± 3.43, respectively, and the matrix effect of these analytes was between 89.05 ± 2.85 and 97.28 ± 0.96. All the above results showed that the consistency, precision, and reproducibility of this method for quantitative analysis of these analytes were acceptable. All the results are exhibited in Table S2.

#### 3.4.5. Stability

At the environmental conditions of room temperature (4 h), 4°C (12 h), −20°C (14 d), and freeze-thaw (3 cycles), the concentration changes of three analytes at low, middle, and high concentration were between −8.32% and 10.03, and the RSD was within 8.46%, which meant these analytes were stable in the above conditions. All the results are exhibited in Table S2.

### 3.5. Pharmacokinetics Results

**Figure 5:**
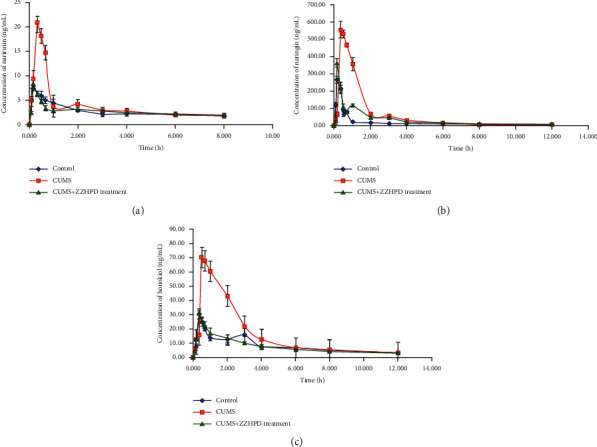
Mean plasma concentration-time profiles of narirutin (a), naringin (b), and honokiol (c) in control, CUMS-induced depression, and CUMS + ZZHPD treatment rats after intragastric administrations of ZZHPD at a dosage of 8.5 g/kg.

The proposed LC-MS/MS method, which was verified in this study, was employed for a comparative pharmacokinetic study of narirutin, naringin, and honokiol in different courses of CUMS-induced liver-stagnation and spleen-deficiency syndrome depressive rats. The concentration-time curves of three ingredients in rats plasma of the control, CUMS, and CUMS + ZZHPD treatment groups are exhibited in Figure 5, while the pharmacokinetic parameters of these chemical compounds are listed in Table 3. Compared with the results of the literature, the pharmacokinetic parameters of narirutin and honokiol in normal rats were different visibly [27], which could be caused by the difference in the dosage of these two ingredients, the herb ratio in ZZHPD and the rats' age. Due to the similar dosage, the pharmacokinetic parameters of naringin, such as *T*_max_ and *C*_max_, were closely [27].

In this study, the *T*_max_ of three chemical constituents in the control, CUMS, and CUMS + ZZHPD treatment groups were as follows (mean ± SD, *n* = 6): narirutin, 0.17 ± 0.00 vs. 0.33 ± 0.00 vs. 0.31 ± 0.07; naringin, 0.17 ± 0.00 vs. 0.36 ± 0.07 vs. 0.17 ± 0.00; honokiol, 0.33 ± 0.00 vs. 0.50 ± 0.00 vs. 0.33 ± 0.00. It could be seen that compared with the control rats, the *T*_max_ of three chemical constituents in CUMS rats was longer, which could be caused by the decreased of the gastrointestinal absorption function of CUMS rats [28, 29], while the call-back of *T*_max_ in CUMS + ZZHPD treatment rats indicated that ZZHPD could relieve depressive symptoms by improving the gastrointestinal absorption function. Similarly, the *C*_max_, AUC_0-t_ , and AUC_0-∞_ of three ingredients in CUMS rats were higher than control rats and then descended in CUMS + ZZHPD treatment rats. Compared with the results of literature, these pharmacokinetic parameters of other ingredients of ZZHPD, including magnolol, hesperidin, neohesperidin, and geniposide, were shown to have the similar variation tendency between rats in control and depression model groups [8]. The change in liver physiological function of CUMS rats may be answerable to the change of these pharmacokinetic parameters [24, 29], while it indicated that ZZHPD could adjust the liver physiological function of CUMS rats. The mechanism of the pharmacokinetic parameter change of these ingredients needs to be revealed in the following researches.

## 4. Conclusion

In this study, a stable, dependable, and rapid method based on UHPLC–MS/MS was employed to uncover the pharmacokinetics of narirutin, naringin, and honokiol in rats of the control, CUMS, and CUMS + ZZHPD treatment groups. The pharmacokinetic processes of these ingredients in different courses of CUMS-induced liver-stagnation and spleen-deficiency syndrome depressive rats after intragastric administration of ZZHPD were compared, resulting in the pharmacokinetic parameters of rats in different physiological and pathological statuses being significantly different. The results could be useful for the dose regimen of ZZHPD for the clinical treatment of depressive disorders.

## Figures and Tables

**Table 1:** The optimized MRM parameters of narirutin, naringin, honokiol, and liquiritin (IS).

Analytes	Q1 (amu)	Q3 (amu)	DP (V)	EP (V)	CE (eV)	CXP (V)
Narirutin	579.3	271.2	−203	−9.0	−33.4	−5.2
Naringin	579.4	271.1	−199	−12.9	−43.7	−23.0
Honokiol	265.3	224.2	−142	−12.4	−30.5	−17.9
Liquiritin (IS)	417.1	255.1	−156	−11.7	−25.8	−10.2

**Table 2:** The linear equation, range, and LLOQ of narirutin, naringin, and honokiol.

Analytes	Linear equation	*r* (*n* = 9)	Range (ng/mL)	LLOQ (ng/mL)	LOD (ng/mL)
Narirutin	*y* = 0.2578*x* − 0.6281	0.9989	1.0–331.2	1.0	0.3
Naringin	*y* = 0.1399*x* − 2.3756	0.9978	3.8–1264.0	3.8	1.3
Honokiol	*y* = 0.1028*x* + 0.0573	0.9978	1.0–330.0	1.0	0.3

**Table 3:** The pharmacokinetic parameters of narirutin, naringin, and honokiol in rats of the control, CUMS, and CUMS + ZZHPD treatment group after oral administration of ZZHPD (8.5 g/kg).

Analytes	Group	*T*_max_ (h)	*C*_max_ (ng/mL)	*t*_1/2_ (h)	AUC_0-t_ (h.ng/mL)	AUC_0-∞_ (h.ng/mL)	CL (L/h/kg)	Vd (L/kg)
Narirutin	Control	0.17 ± 0.00	7.33 ± 0.46	3.17 ± 0.47	21.95 ± 1.51	26.58 ± 2.85	54.55 ± 6.06	247.06 ± 21.90
	CUMS	0.33 ± 0.00 ^*∗∗∗*^	21.03 ± 0.95^*∗∗∗*^	2.32 ± 0.38^*∗∗*^	31.22 ± 2.60^*∗∗∗*^	34.44 ± 3.62^*∗∗*^	42.06 ± 4.34^*∗∗*^	139.53 ± 16.08^*∗∗∗*^
	CUMS + ZZHPD treatment	0.31 ± 0.07	8.34 ± 0.95^###^	6.01 ± 1.40^###^	21.23 ± 0.54^###^	35.93 ± 5.45	40.61 ± 5.22	343.68 ± 26.26^###^

Naringin	Control	0.17 ± 0.00	268.82 ± 19.96	2.66 ± 0.18	251.16 ± 16.78	264.64 ± 19.49	81.09 ± 6.07	309.90 ± 16.32
	CUMS	0.36 ± 0.07^*∗∗∗*^	576.90 ± 45.37^*∗∗∗*^	2.10 ± 0.10^*∗∗∗*^	813.79 ± 27.30^*∗∗∗*^	825.82 ± 27.38^*∗∗∗*^	25.89 ± 0.88^*∗∗∗*^	78.29 ± 4.85^*∗∗∗*^
	CUMS + ZZHPD treatment	0.17 ± 0.00^###^	366.67 ± 25.99^###^	2.81 ± 0.43^##^	434.98 ± 26.10^###^	458.33 ± 30.64^###^	46.78 ± 3.19^###^	189.15 ± 25.50^###^

Honokiol	Control	0.33 ± 0.00	29.72 ± 3.04	5.74 ± 1.35	96.11 ± 7.33	123.82 ± 18.11	35.46 ± 5.54	285.56 ± 34.26
	CUMS	0.50 ± 0.00	70.13 ± 2.38 ^*∗∗∗*^	3.73 ± 0.63 ^*∗∗*^	193.74 ± 13.49 ^*∗∗∗*^	211.13 ± 20.18 ^*∗∗∗*^	20.57 ± 2.13^*∗∗∗*^	109.32 ± 8.81^*∗∗∗*^
	CUMS + ZZHPD treatment	0.33 ± 0.00	30.80 ± 3.63^###^	4.37 ± 0.31^###^	92.82 ± 7.03^###^	108.30 ± 8.83	40.00 ± 3.27^###^	251.88 ± 21.01^###^

*Note.* ^∗∗^ *p* < 0.01, ^*∗∗∗*^*p* < 0.001, compared with control group;^##^*p* < 0.01,^###^*p* < 0.001, compared with CUMS group (mean ± SD, *n* = 6).

## Data Availability

The data used to support the findings of this study are included within the article and the supplementary information file.

## Abbreviations

ZZHPD: Zhi-Zi-Hou-Po decoction
PX: Paroxetine hydrochloride
CUMS: Chronic unpredictable mild stress
TCMF: Traditional Chinese medicine formula
SPT: Sucrose preference test
FST: Forced swimming test
OFT: Open-field test
IS: Internal standard
LLOQ: Lower limit of quantification
LOD: Limit of detection.

## Supplementary Materials

Supplementary Materials Text S1: the conversion rule of oral dose of ZZHPD and PX. Figure S1: the animal experimental design of this study (10 weeks). Figure S2: the standard curves of narirutin (a), naringin (b), and honokiol (c) (*r* > 0.99). Table S1: the intraday and interday precision, accuracy, extraction recovery, and matrix effects of three analytes at low, middle, and high concentration. Table S2: the stability of three analytes at low, middle, and high concentration.
