# Supplementary material for: Comparative Pharmacokinetic Study of Three Major Bioactive Components in the Normal and Different Courses of Liver-Stagnation and Spleen-Deficiency Syndrome Depressive Rats after Intragastric Administration of Zhi-Zi-Hou-Po Decoction
Source: J Anal Methods Chem. 2022 Mar 24;2022:8657616. doi: 10.1155/2022/8657616 (PMC8970962; doi:10.1155/2022/8657616)
Supplement: Supplementary Materials — Text S1: the conversion rule of oral dose of ZZHPD and PX. Figure S1: the animal experimental design of this study (10 weeks). Figure S2: the standard curves of narirutin (a), naringin (b), and honokiol (c) (r > 0.99). Table S1: the intraday and interday precision, accuracy, extraction recovery, and matrix effects of three analytes at low, middle, and high concentration. Table S2: the stability of three analytes at low, middle, and high concentration. [file 8657616.f1.pdf]

Comparative pharmacokinetic study of three major bioactive components in normal and different course of liver-stagnation and spleen-deficiency syndrome depressive rats after intragastric administration of Zhi-Zi-Hou-Po decoction

Kaiwen Luo*, Mengdie Wang, Xiangxiang Pan, Yadong Xing

School of Pharmacy, Bengbu Medical University, Bengbu, 233030, China

Corresponding author:

Dr. Kaiwen Luo

School of Pharmacy, Bengbu Medical University

Bengbu, 233030, China

Tel: +86 0552-3175452

E-mail addresses: kevinrowe@163.com

Supplemental File

Text S1: The conversion rule of oral dose of ZZHPD and PX.

As recorded in *Shang-Han-Lun* (*Treatise on Febrile Caused by Cold*), the oral dose of ZZHPD is 81.4 g/d (ZZ 9 g, HP 62.4 g and ZS 10 g) for a human being. In consideration of the average weight of human is 60 kg, the oral dose is 1.36 g/kg/d. After multiply by the reduction coefficient, which is 6.3, between human and rat, the oral dose for rat is 8.5 g/kg/d.

The recommended oral dose of paroxetine for human is 20 mg/d, then the oral dose for rat is 2.1 mg/kg/d according to the same conversion way.

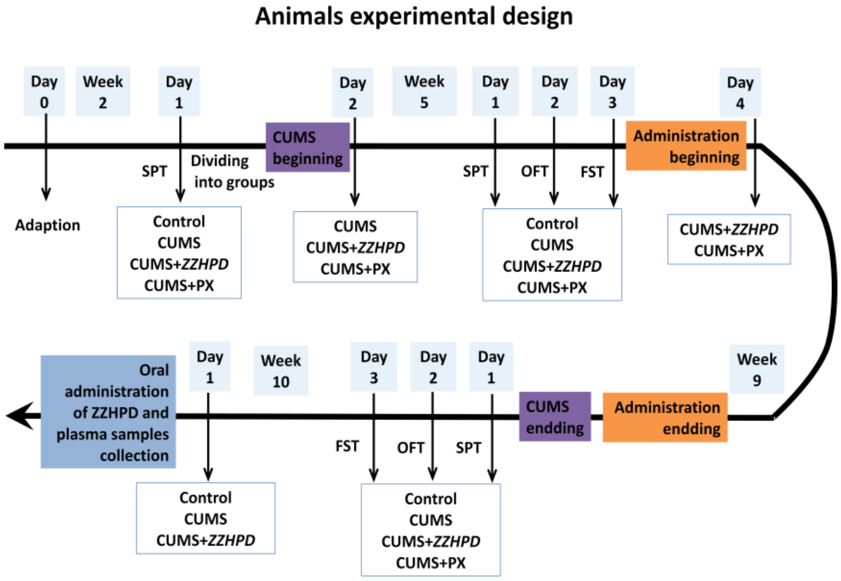

FIGURE S1. The animal experimental design of this study (10 weeks).

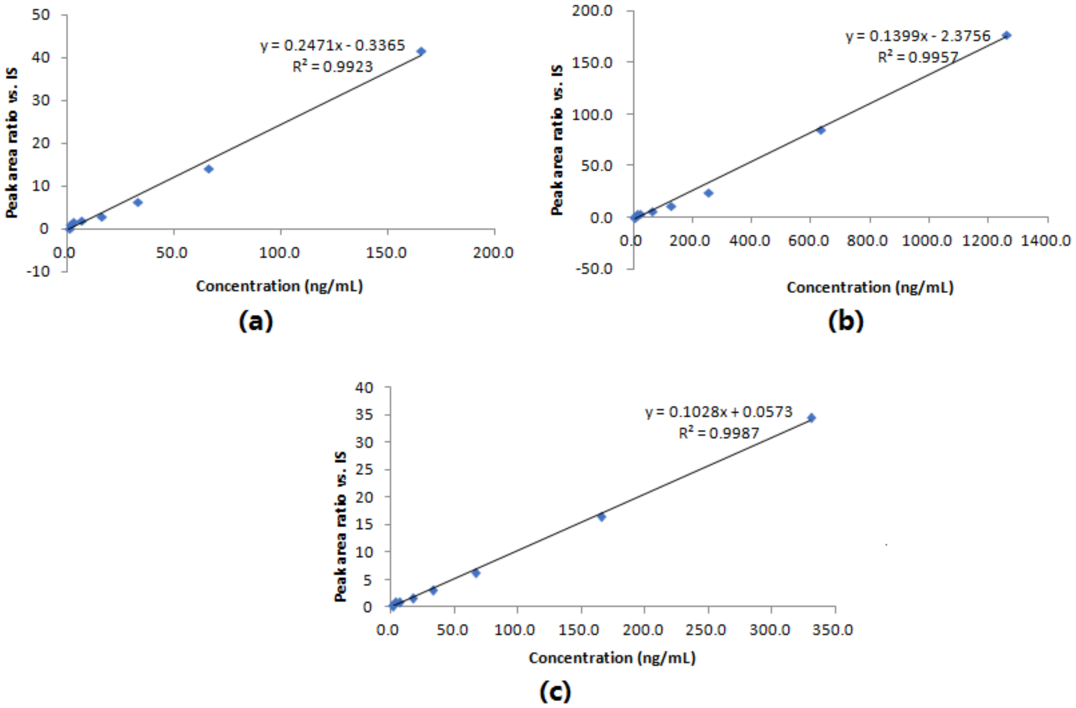

FIGURE S2: The standard curves of narirutin (a), naringin (b) and honokiol (c) ($r > 0.99$).

TABLE S1: The intra-day and Inter-day precision, accuracy, extraction recovery and matrix effects of three analytes at low, middle and high concentration.

Analytes	Concentration (ng/mL)	Intra-day precision RSD (%)	Inter-day precision RSD (%)	Accuracy RE (%)	Extraction recovery (%, mean ± SD)	Matrix effects (%, mean ±SD)
Narirutin	2.5	0.45	1.31	3.45	92.25±4.21	94.87±2.17
	20.0	0.96	0.75	-4.16	93.56±1.79	95.67±3.01
	265.0	1.39	2.11	6.16	98.23±5.36	95.06±1.29
Naringin	9.5	0.59	0.73	7.35	89.62±2.76	89.05±2.85
	76.0	0.28	0.33	-5.21	90.72±3.75	93.05±0.87
	1011.2	1.57	2.01	7.91	99.69±5.56	97.28±0.96
Honokiol	2.5	2.39	2.14	4.83	91.92±1.03	93.72±0.56
	20.0	1.56	1.74	8.14	95.12±2.06	93.52±3.15
	264.0	2.17	1.78	-7.28	104.71±3.43	89.51±3.25

TABLE S2: The stability of three analytes at low, middle and high concentration.

Analytes	Concentration (ng/mL)	Stability at room temperature for 4 h		Stability in autosampler for 12 h		Stability of repeated freeze-thaw for three times		Stability at -20℃ for 14 days	
		RE (%)	RSD (%)	RE (%)	RSD (%)	RE (%)	RSD (%)	RE (%)	RSD (%)
Narirutin	2.5	5.98	3.71	8.10	2.50	9.38	4.82	-6.11	5.92
	20.0	7.81	2.53	10.03	2.97	-8.32	3.72	9.32	6.21
	265.0	-5.32	2.11	-4.02	1.18	-3.21	1.07	1.05	4.02
Naringin	9.5	6.01	4.78	-5.22	1.57	8.93	4.19	5.32	7.29
	76.0	-4.01	1.76	5.91	3.50	4.73	2.89	-3.02	5.83
	1011.2	6.38	0.92	4.39	0.31	5.22	0.31	4.61	3.49
Honokiol	2.5	9.96	4.21	-3.29	0.90	3.02	3.78	7.65	8.46
	20.0	-7.53	2.41	4.41	0.04	5.92	2.15	-3.87	5.15
	264.0	3.29	1.55	-2.13	1.90	1.01	2.37	3.69	2.44
